# Supplementary figures and images for: A Bayesian approach for fitting and comparing demographic growth models of radiocarbon dates: A case study on the Jomon-Yayoi transition in Kyushu (Japan)
Source: PLoS One. 2021 May 19;16(5):e0251695. doi: 10.1371/journal.pone.0251695 (PMC8133439; doi:10.1371/journal.pone.0251695)

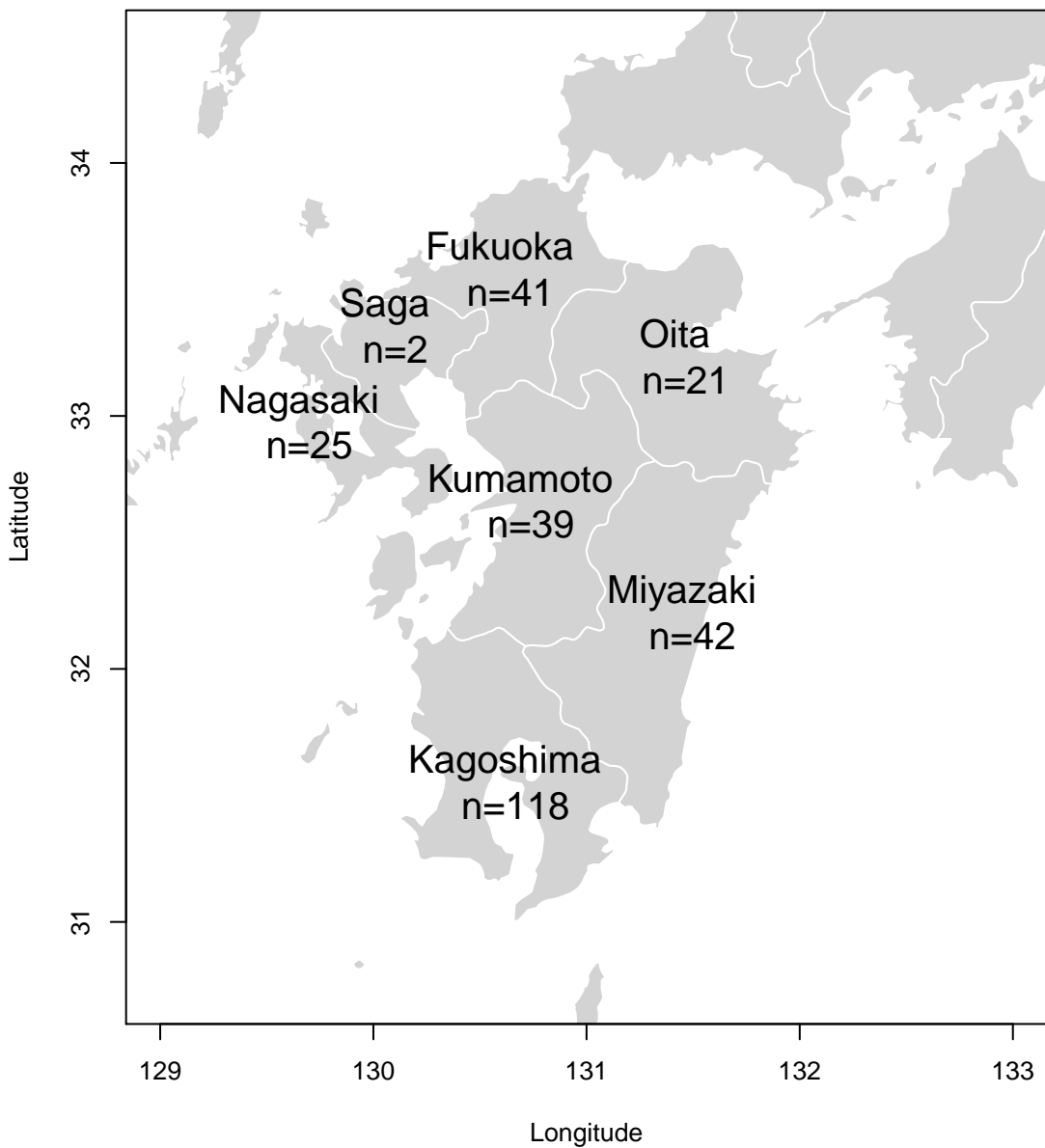

Supplement: S1 Fig — Base maps made with Natural Earth. (PDF) [file pone.0251695.s001.pdf]

Setting 1

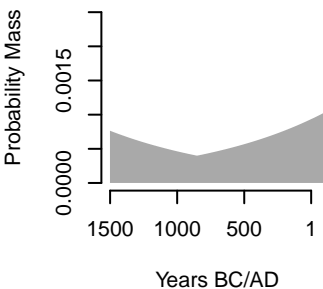

Setting 4

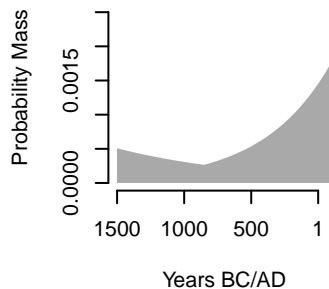

Setting 7

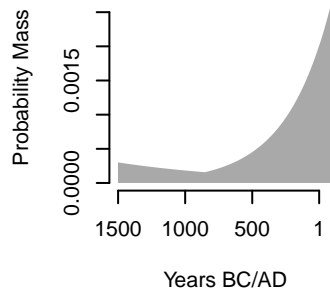

Setting 2

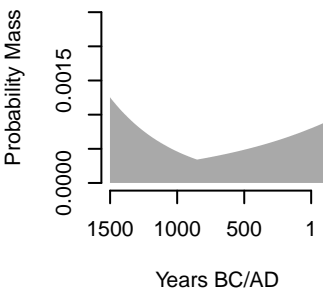

Setting 5

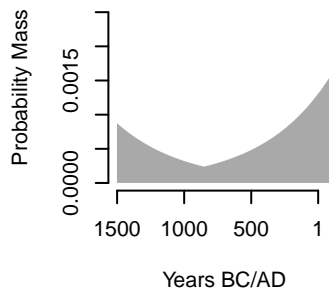

Setting 8

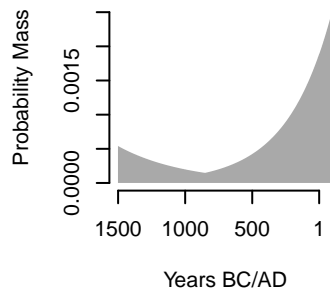

Setting 3

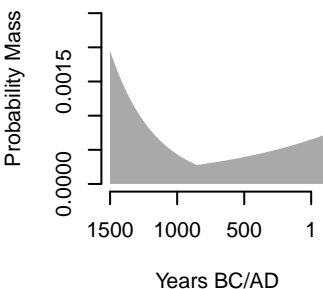

Setting 6

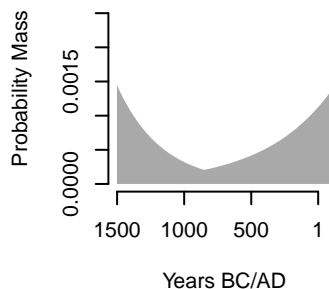

Setting 9

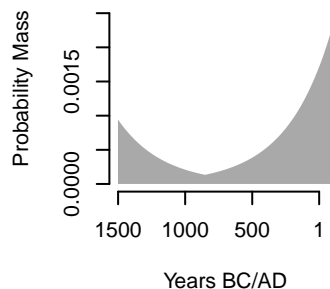

Supplement: S2 Fig — (PDF) [file pone.0251695.s002.pdf]

Probability

**m1**

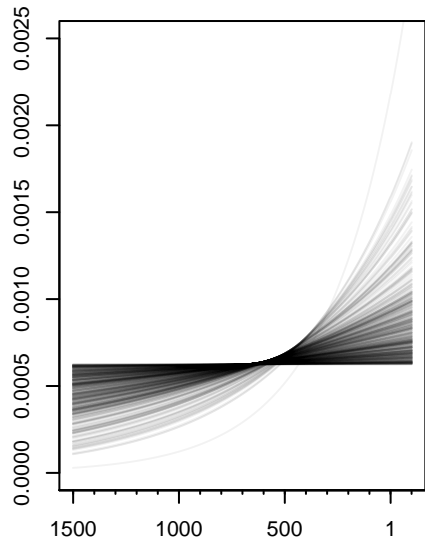

Years BC/AD

Probability

**m2**

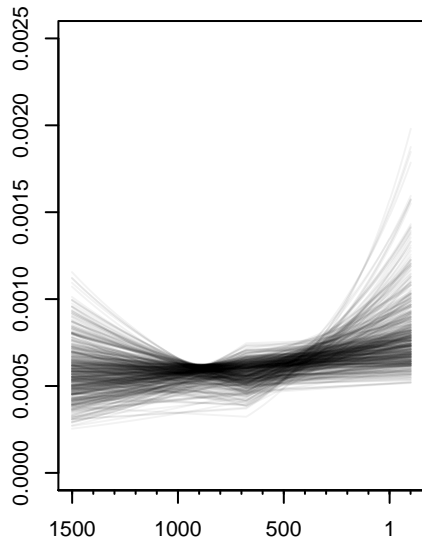

Years BC/AD

Probability

**m3**

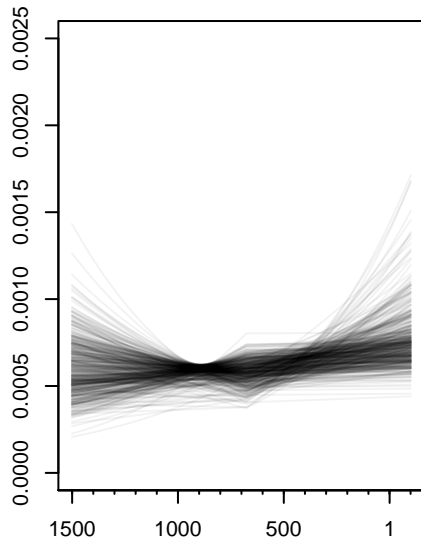

Years BC/AD

Supplement: S3 Fig — (PDF) [file pone.0251695.s003.pdf]

**Chain 1**

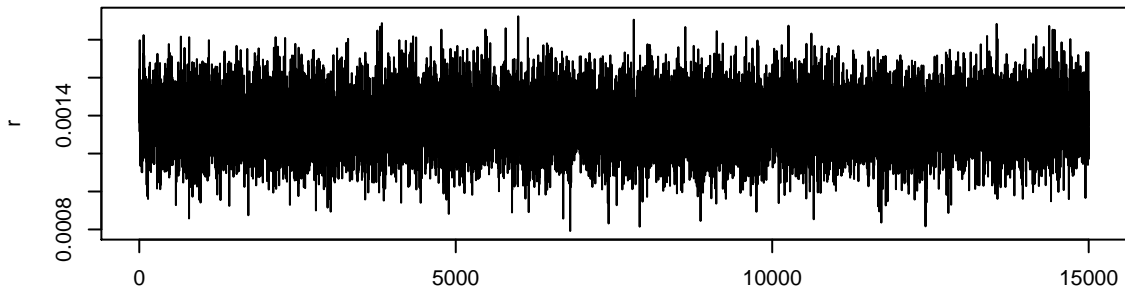

MCMC Sample

**Chain 2**

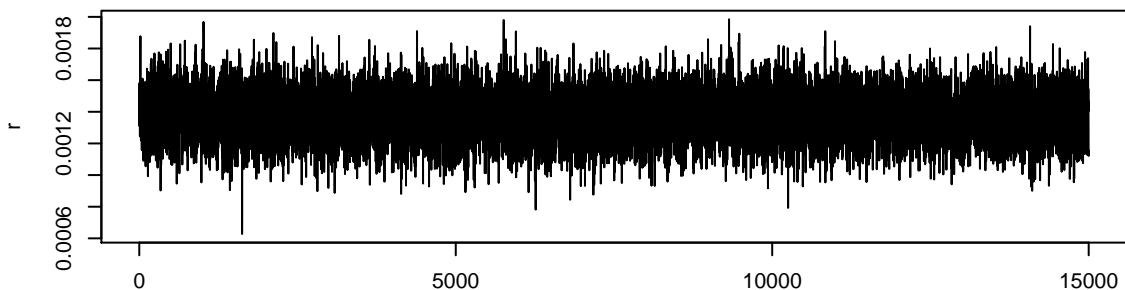

MCMC Sample

**Chain 3**

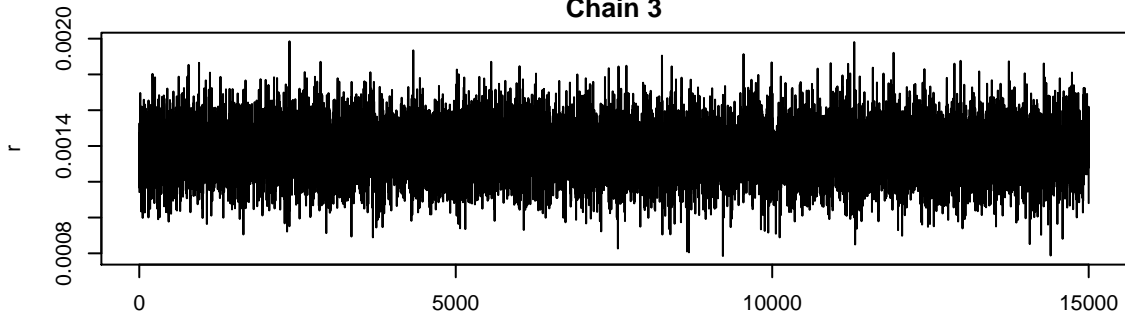

MCMC Sample

Supplement: S4 Fig — (PDF) [file pone.0251695.s004.pdf]

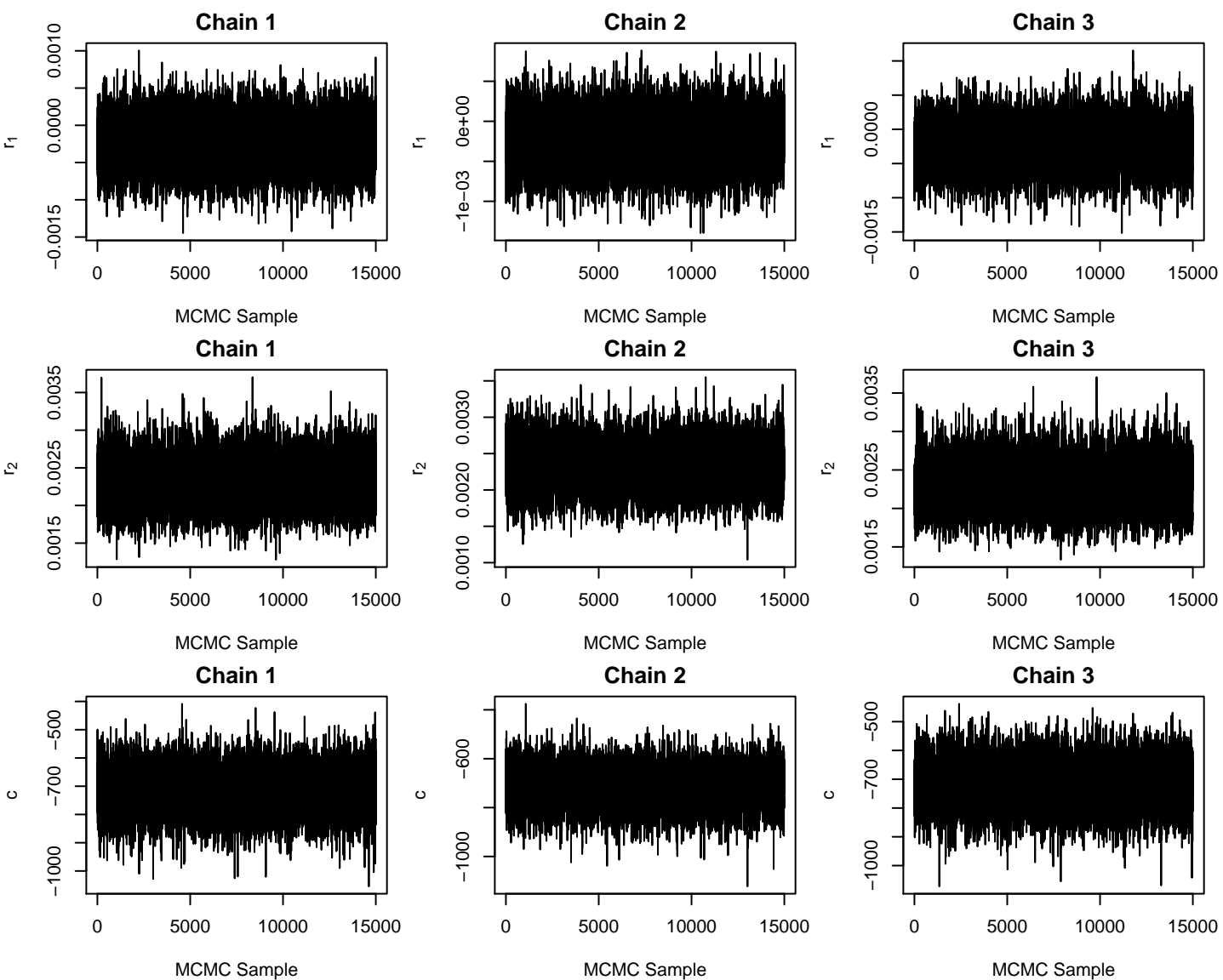

Supplement: S5 Fig — (PDF) [file pone.0251695.s005.pdf]

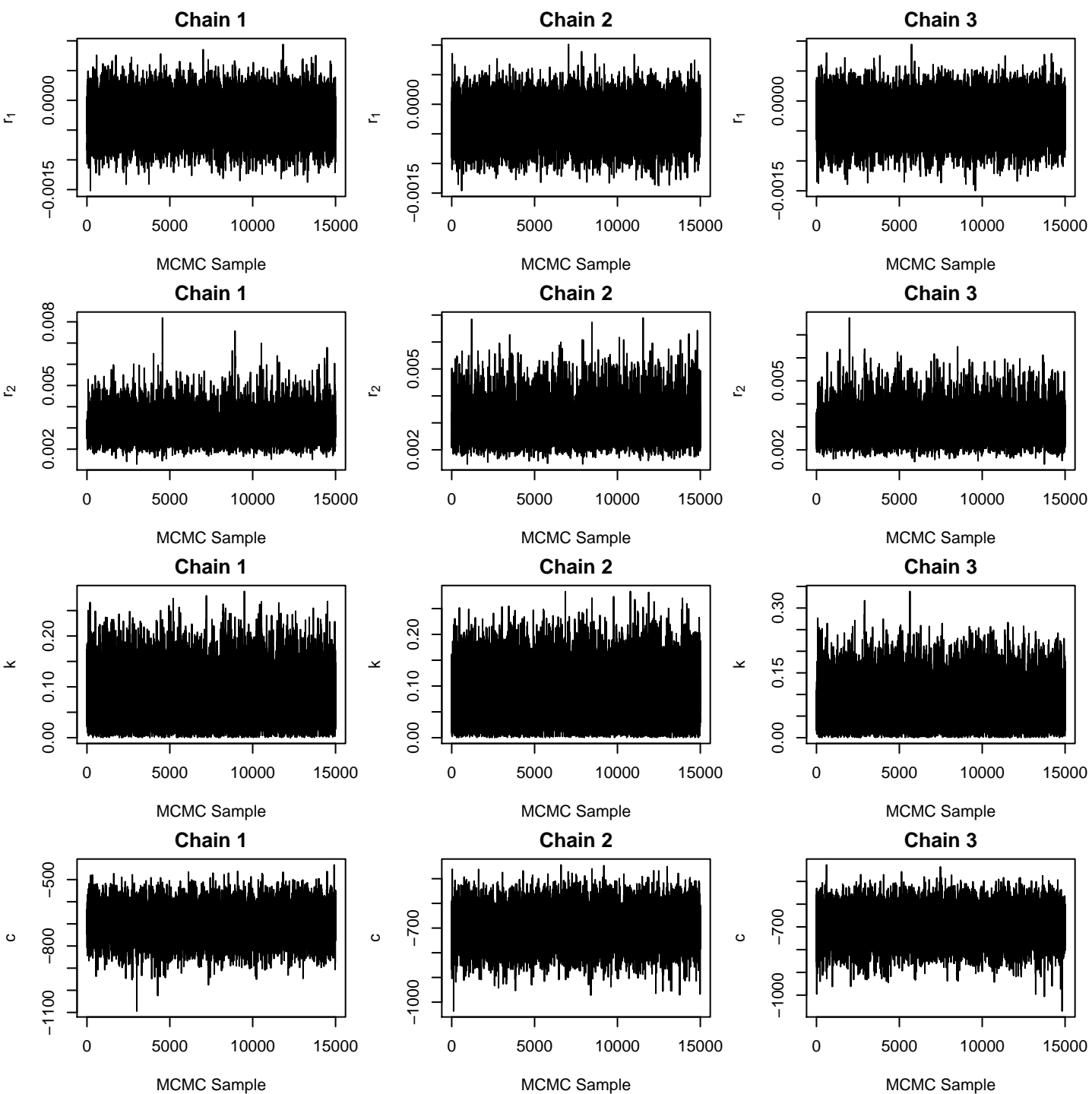

Supplement: S6 Fig — (PDF) [file pone.0251695.s006.pdf]

### m2 changepoint vs earliest occurence of rice

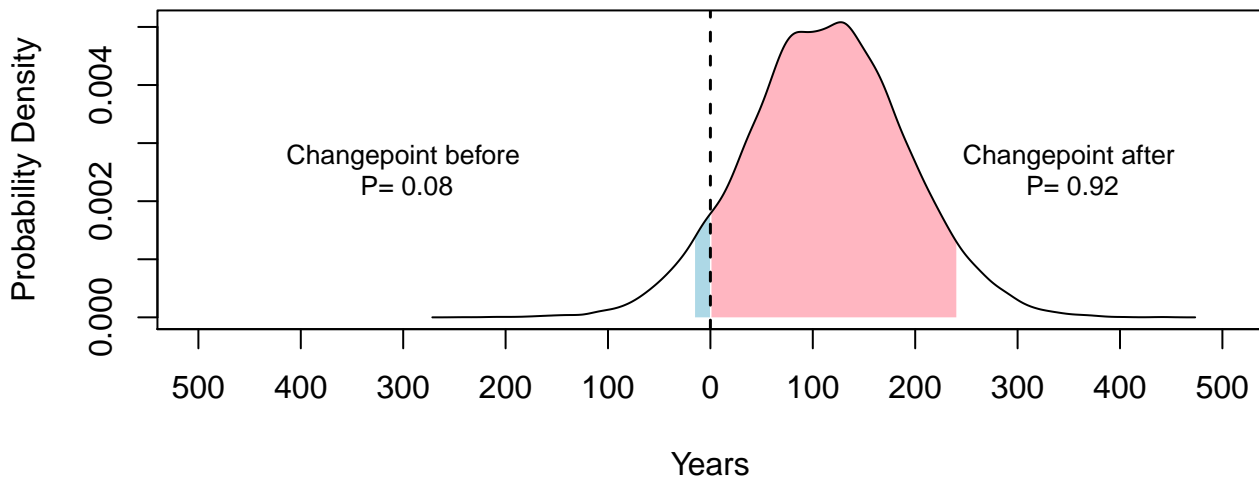

### m3 changepoint vs earliest occurence of rice

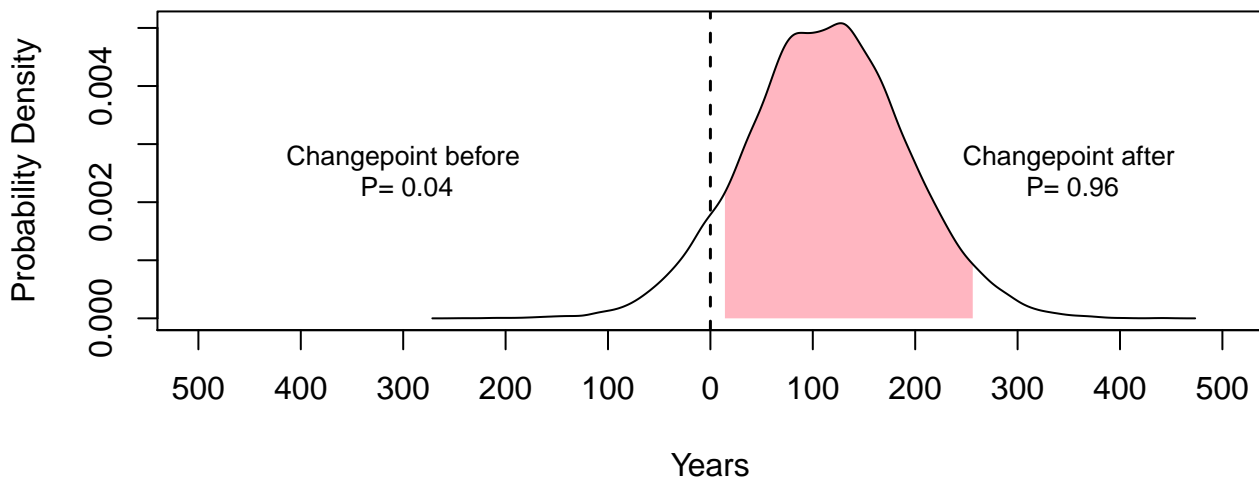

Supplement: S7 Fig — Differences were computed by comparing 15,000 random pairs of posterior samples from the two events. The highlighted region represents the 90% HPD density interval. Rice dates were calibrated using IntCal20 and combined using the R_Combine function in OxCal (via the OxCAAR R package). (PDF) [file pone.0251695.s007.pdf]
