# Supplementary material for: A Bayesian approach for fitting and comparing demographic growth models of radiocarbon dates: A case study on the Jomon-Yayoi transition in Kyushu (Japan)
Source: PLoS One. 2021 May 19;16(5):e0251695. doi: 10.1371/journal.pone.0251695 (PMC8133439; doi:10.1371/journal.pone.0251695)
Supplement: Attachment — Submitted filename: Response to Reviewers.pdf [file pone.0251695.s009.pdf]

1.JOURNAL REQUIREMENTS

1.1 Permits

We added a statement to make clear that no permits were required for the study at lines 515-516)

1.2 'We note that S1 Figure in your submission contains map images which may be copyrighted.'

We have revised figure S1 following the journal recommendation using base maps from Natural Earth via the *rnaturalearth* R package and acknowledged this also in the figure caption (line 1160-1161)

2.REVIEWERS COMMENTS

2.1 Reviewer 1

Not applicable (no issues to be addressed)

2.2 Reviewer 2

2.2.1 Lack of discussion pertaining to population ecology theory and more broader theoretical implications of SPDs.

We agree with the reviewer and the editor that these are important and topical aspects of prehistoric demography. We revised the manuscript by: a) introducing a brief discussion on growth models in the early part of the manuscript (lines 113-116) ; b) providing a more detailed theoretical justification of our three models for the case study (lines 541-542, 546-549, and 558-561); and c) expanding discussion for future direction by emphasising a stronger link between theoretical population ecology models and the specific empirical challenges of radiocarbon data (lines 741-744, 772-773). We felt however that a more comprehensive discussion on theoretical population ecology model is somewhat out of the scope of the paper so we limited the discussion to only key points.

2.2.2 'Bayesian statistics are predicated on the ability to use logically-sound prior information to formalize the radiocarbon calibration process (cf. Buck and Meson, World Archaeology, 2015). There is a specific philosophy to Bayesian approaches (Buck and Meson, 2015), which the authors do not consider.'

We are not entirely sure what specific aspects of the Bayesian philosophy we have not accounted for, but have made a more explicit discussion on the choice of weakly informative priors (lines 568-572) and referenced the 'what if' experiments discussed in Buck and Meson 2015 in relation to our experiment 4 (lines 761-765).

2.2.3 '[...] I'm wondering why the r parameters used in the simulations are not similar to the prior produced rates they cite for the Jomon-Yayoi transition? [...]'

We did use comparable rates (in terms of order of magnitude) to existing studies based on other proxies. We believe this confusion was raised by the fact that we provided growth rates both in raw values and in %. To ensure that it was clear we added an extra sentence in lines 505-507.

2.2.4 'Also, the authors do not explain their rationale for radiocarbon sample selection criteria excluding standard errors larger than 100 years. This seems awfully precise compared to standard practices that exclude errors <200 years or <300 years. I'm not saying the authors should not do this, but that they simply need to justify why they do.'

The main rationale for using dates with smaller standard errors was dictated by our objective to identify with better precision the timing of the changepoint by minimising the measurement error. There is however no formal justification, i.e. this is as arbitrary as 200 and 300 years. We briefly specify our logic in lines 517-518.

2.2.5 'Certain techniques are not clearly explained to the reader. An example of this is the Gelman-Rubin's convergence diagnostic, or, the WAIC. '

We briefly explain both concepts in lines 594-598 and 607-609.

2.2.6 On page 20 the authors note that they did not try running a larger number of MCMC iterations, but then they state that "the results do seem to suggest that an ideal minimum sample size is specific to the model and its parameters". This is confusing to read, and raises a red-flag due to the lack of elaboration. I feel like there needs to be more sensitivity analysis in this part of the work.

We edited the sentence to make clear our point lines 636-641.

2.3 Reviewer 3

2.3.1 '[...] sentence covering lines 479 to 480 on page 19 is confusing'

We edited the sentence to make our statement clear (lines 622-625)

2.3.2 '[...] I would be interested to see how well the methods hold up with radiocarbon dates that have a higher error, or for older parts of the calibration curve with more error. However, given the depth of what is covered already in the paper, I understand that it would be unnecessary to include that within this specific piece of research.'

This is an important point that cannot be simply addressed by a comprehensive study as the number and combination of variables (sample size, measurement & calibration error, idiosyncrasies of the calibration curve, choice of priors, the complexity and the number of the parameters of the growth model) is extremely large. We recommend users to run test simulations (as we did in experiment 4) for their specific case to evaluate the inferential

power of this approach. As this is an important point, we added an extra sentence in lines 761-765 to stress this.

3.OTHER CHANGES IN THE MANUSCRIPT

We report the following additional changes in the manuscript:

- Figure 6's point size has been slightly reduced to improve readability
- Figure 3's y-axis label was missing and has now been added.
- We added an acknowledgement section.
- We slightly expanded our discussion of the result of the case study in lines 826-840, mostly to clarify the potential impact of repeated migration waves to the population dynamics.
- We fixed some minor typos and made some minor stylistic changes (See track changes).
- We added the following references: Marwick 2017, Buck and Meson 2015, Turchin 2003 and Kawahata et al 2016.
