# Supplementary material for: A Bayesian approach for fitting and comparing demographic growth models of radiocarbon dates: A case study on the Jomon-Yayoi transition in Kyushu (Japan)
Source: PLoS One. 2021 May 19;16(5):e0251695. doi: 10.1371/journal.pone.0251695 (PMC8133439; doi:10.1371/journal.pone.0251695)
Supplement: Attachment — Submitted filename: Review_PONE_D_21_02107.docx [file pone.0251695.s008.docx]

Review of PONE-D-21-02107

“A Bayesian approach for fitting and comparing demographic growth models of radiocarbon dates: a case study on the Jomon-Yayoi transition in Kyushu (Japan)”

Yet again, the authors have produced an important tool that advances the use of radiocarbon SPDs as a proxy measure of prehistoric demography. This is possibly their most creative and cutting edge of all their great contributions to SPD research. It certainly merits publication in a high impact journal such as PlosOne. However, while the basic math and rationale makes sense to me, I have some issues with the lack of theoretical discussion in this paper. This is reflected in their general framing of the problem they address. While they rightly frame the problem in terms of the use of SPDs, they do not discuss the actual theoretical issues involved in using an SPD as a relative, coarse-grained measure of prehistoric demography. A persistant problem for me with the Null Hypothesis Statistical Testing approach and other recent advances to SPD analysis has been the lack of consideration of these null models for what they mean in the broader context of Population Ecology Theory (cf. Turchin, Complex Population Dynamics, 2003). The exponential and logistic models have very specific general theory attached to them that the authors of this paper do not consider. Bayesian statistics are predicated on the ability to use logically-sound prior information to formalize the radiocarbon calibration process (cf. Buck and Meson, World Archaeology, 2015). There is a specific philosophy to Bayesian approaches (Buck and Meson, 2015), which the authors do not consider.

What is the basic math and theory behind an exponential growth model? Behind a Double-exponential model? I worry that, while this is another excellent technical contribution to the use of SPDs, there’s still a lack of general discussion of what these various growth models actually mean for general population ecology theory. Technical advances are important to ensure researchers do not misinterpret trends in radiocarbon SPDs. But, what about the broader theoretical implications of how these technical advances might be taken up and implemented by other researchers? I know that the authors are also skilled at theory building, which is why I challenge them to more fully articulate the growth models they employ within their new method within the broader philosophies of Bayesian statistics, and, General Population Ecology theory. This paper has the potential to be a game-changer, especially compared to the other recent advances that they mention, which in my opinion, are still vague on these general theoretical levels. New technical advances are one thing, but logical clarity in their background theory ultimately enhances their proper use by other researchers down the line. I would be very curious how Bayesian pioneers such as Caitlin Buck, or Population Ecologists such as Peter Turchin, would read this paper. In my opinion, the discussion of the different growth models used should be discussed much earlier in the paper than on page 17. And, when the authors do this, the text is very dense with math, but not accompanied by general theory. This potentially limits the impact of this paper, as many readers might not understand what’s going on. Which, increases the potential that users might mis-use the technical advances down the line. The opening part of the discussion is on-point, and articulates the current problems with NHST very well. However, this kind of discussion must be integrated in the initial framing of the problem in the introduction. It’s quite frustrating to me to read on page 24 that “Our method can be easily extended to other growth models”. What are these specific growth models? Are they based in the general theory of population ecology?

As a more specific question, I’m wondering why the *r* parameters used in the simulations are not similar to the prior produced rates they cite for the Jomon-Yayoi transition? I understand that these were produced using other proxies, but actually, this helps to strengthen the logic of the prior parameters built into the simulation experiments. These of course seem abnormally high to me, and the parameters the authors use in this paper are closer to realistic (although, they’re very low). But, I would be curious what would result from the authors using a wider range of parameter values for *r*.

Also, the authors do not explain their rationale for radiocarbon sample selection criteria excluding standard errors larger than 100 years. This seems awfully precise compared to standard practices that exlude errors <200 years or <300 years. I’m not saying the authors should not do this, but that they simply need to justify why they do.

Certain techniques are not clearly explained to the reader. An example of this is the Gelman-Rubin’s convergence diagnostic, or, the WAIC. What do these actually do? The authors mention at the top of the paper the insufficiency of AIC or BIC approaches, and propose that WAIC is better. But, there is no general discussion from the start of what that is. The authors cannot expect the readers to be as smart and savvy with statistical information criterion as they are. Different information criterion are actually very new to archaeology, and much of the field is still not using them to evaluate the performance of different models. I say all this because, as I mentioned above, this paper has the potential to be a ‘game-changer’. But, if the content is too dense and not made easily understandable to a wide-audience, then it risks: 1) not being used by as many researchers as it deserves; 2) being mis-used and mis-interpreted by researchers down the line.

On page 20 the authors note that they did not try running a larger number of MCMC iterations, but then they state that “the results do seem to suggest that an ideal minimum sample size is specific to the model and its parameters”. This is confusing to read, and raises a red-flag due to the lack of elaboration. I feel like there needs to be more sensitivity analysis in this part of the work.

Overall, this is a technically creative, savvy, and very important contribution to SPD research. I commend the authors on their continued important contributions to this field. I firmly believe that the overall contribution of this paper can be enhanced by doing what other SPD advances have not done: actually couching the rationale behind the technical advances within 1) Population Ecology theory, and 2) Bayesian philosophy.

Sincerely,

Erick Robinson

Anthropology Program, Utah State University
